# Local and global contributions to hemodynamic activity in mouse cortex

**DOI:** 10.1152/jn.00125.2016

**Published:** 2016-03-16

**Authors:** M. Andrea Pisauro, Andrea Benucci, Matteo Carandini

**Affiliations:** Institute of Ophthalmology, University College London, London, United Kingdom

**Keywords:** brain imaging, hemodynamic activity, neurovascular coupling

## Abstract

Imaging techniques such as functional magnetic resonance imaging seek to estimate neural signals in local brain regions through measurements of hemodynamic activity. However, hemodynamic activity is accompanied by large vascular fluctuations of unclear significance. To characterize these fluctuations and their impact on estimates of neural signals, we used optical imaging in visual cortex of awake mice. We found that hemodynamic activity can be expressed as the sum of two components, one local and one global. The local component reflected presumed neural signals driven by visual stimuli in the appropriate retinotopic region. The global component constituted large fluctuations shared by larger cortical regions, which extend beyond visual cortex. These fluctuations varied from trial to trial, but they did not constitute noise; they correlated with pupil diameter, suggesting that they reflect variations in arousal or alertness. Distinguishing local and global contributions to hemodynamic activity may help understand neurovascular coupling and interpret measurements of hemodynamic responses.

in experiments based on functional magnetic resonance imaging (fMRI) or optical imaging, neural signals are inferred through measurements of hemodynamic activity. This activity reflects neural signals because of neurovascular coupling, which increases the volume of blood flowing into the activated area (Attwell et al. 2010). Despite its slower temporal dynamics, hemodynamic activity is thought to be roughly proportional to the underlying neural signals, providing effectively a blurred version of those signals (Boynton et al. 1996; Cardoso et al. 2012; Kahn et al. 2011).

However, functional imaging measurements reveal that hemodynamic activity in sensory brain regions is highly variable across stimulus repetitions (Fox and Raichle 2007; Grinvald et al. 1986). This variability may reflect fluctuations in the underlying neural activity, in neurovascular coupling, or in other sources of hemodynamic activity. It is typically thought to constitute “noise,” because it is not predictable from the sensory stimuli. Indeed, techniques for estimating neural activity from fMRI signals typically prescribe methods so that this “noise” can be discarded (Engel et al. 1994; Friston et al. 1994; Logothetis 2008; Sereno et al. 1995).

However, the apparently variable hemodynamic activity may not constitute noise: it may relate deterministically to physiological and behavioral factors such as brain state and alertness. For instance, optical imaging in visual cortex of behaving monkeys revealed a modulation of hemodynamic signals that matches the temporal structure of the behavioral task (Cardoso et al. 2012; Sirotin and Das 2009). This modulation may reflect task-related changes in brain state, e.g., in alertness.

To study fluctuations in hemodynamic activity, we used optical imaging in the visual cortex of awake mice. To isolate the component of hemodynamic activity that reflects local neural responses, we presented visual stimuli that move across the visual field (Engel et al. 1994; Kalatsky and Stryker 2003). The stimuli elicit traveling waves of local neural responses, and thus traveling waves in the associated hemodynamic activity. The remaining component of hemodynamic activity was composed of large fluctuations that were synchronous across the visual cortex. These global fluctuations were independent of the stimulus, but they correlated with fluctuations in pupil diameter, suggesting that they reflect variations in alertness.

## MATERIALS AND METHODS

Experiments were conducted under licenses released by the Home Office following institutional ethics review, according to the UK Animals Scientific Procedures Act (1986 Amendment Regulations 2012).

### 

#### Initial surgery.

Mice (8–20 wk old, male, C57BL/6J) were anesthetized and implanted with a head post and a window to provide optical access to the right visual cortex through thinned skull. The head post and optical chamber were fixed to the bone with dental cement (Sun Medical). We used *N*-butyl cyanoacrylate (Vetbond; 3M) to glue a 5-mm-diameter glass coverslip inside the chamber. This preparation allowed imaging for up to 3 mo.

#### Imaging.

After recovery (3–4 days after surgery) mice were head-fixed on a ball suspended on a stream of air (Harvey et al. 2009), where they were free to run or stay stationary. After 2–3 sessions of acclimatization, we began imaging sessions, each lasting typically ∼1 h. We illuminated the cortex with two green light-emitting diodes (LEDs; 530 ± 20 nm, M530F1; ThorLabs) focused with collimators (F280FC-A; ThorLabs). We imaged the cortex using a CMOS camera (MV-D1024E-160; Photonfocus) or an sCMOS camera (PCO.edge; PCO) through a ×1.6 objective lens (Planapo-M; Leica). We focused the camera ∼200 μm beneath the superficial blood vessels and used a black cone to shield the lens from the light of the monitors. We typically acquired images at 10 Hz, occasionally 25 Hz.

#### Visual stimuli.

Stimuli were presented on three gamma-corrected LCD monitors (NEC Multisync LCD 2190UXp or HannsG HW191, refresh rate 60 Hz) arranged in a semicircle (radius ∼30 cm) around the mouse. Stimuli were moving flickering bars (Kalatsky and Stryker 2003), typically 1° wide, drifting slowly and periodically (0.10–0.12 Hz) across 90° (azimuth)/70° (elevation) of contralateral visual field for 5–10 cycles (for a duration of 50–100 s). We typically presented three to five repetitions of two such stimuli (vertical or horizontal) running in opposite directions in random order. The bars reversed in contrast at 2 Hz, at 100% contrast, while mean luminance was kept constant. We performed 42 such experiments on 13 mice.

#### Local and global components of hemodynamic activity.

The concatenated responses to these stimuli yielded a two-dimensional (2-D) function of space *s* and time *t*, *A*(*s*, *t*), where *s* encompasses both coordinates *x*, *y*. To fit this matrix with a space-time separable model (see *Eq. 1*), we used singular value decomposition and obtained functions of space, *F*(*s*), and time, *G*(*t*). We named *R*(*s*, *t*) the residual, i.e., the portion of the data that is not explained by the model. We then normalized both the separable component and its residual by the global image, *F*(*s*). The result (see *Eq. 2*) yields a global component, *G*(*t*), and a local component, *L*(*s*, *t*) = *R*(*s*, *t*)/*F*(*s*).

#### Eye tracking.

We recorded eye movements and pupil dilation in 26 experiments on 12 mice. We imaged the left eye using a CCD camera (DMK 21BU04.H; Imaging Source) and illumination from two infrared LEDs (850 ± 20 nm, SLS-0208-D; Mightex). Images were sampled at 15 Hz or higher. We detected blinks by correlating individual frames to the average frame; we then discarded blinks frames. After cropping a region of interest (ROI) around the pupil, we *1*) normalized the images to their maximum intensity, *2*) filtered them spatially (Gaussian bandpass filter), *3*) enhanced the contrast (MATLAB function imadjust), *4*) thresholded them to find the pupil, and *5*) removed spurious pixels and filled gaps in the pupil (functions bwareaopen and imclose). We then fitted an ellipse around the pupil (function regionprops) and used its center to compute eye movements and its area as a measure of pupil size. We converted pixel coordinates to degrees of visual field using a model of the C57BL/6J mouse eye (Sakatani and Isa 2004) based on anatomic measurements (Remtulla and Hallett 1985). We measured pupil dilation as the square root of the ellipse area, relative to the average.

#### Correlations.

In 17 experiments on 10 awake mice, we computed correlation between the pupil diameter *d*(*t*) and the global component *G*(*t*) of hemodynamic responses. We downsampled the traces to a common rate (1 Hz) and computed the kernel *k*(τ) that best predicts trace *G*(*t*) by convolution with *d*(*t*):

$$G_{est}\left(t\right)=\left[k\cdot d\right]\left(t\right)+\varepsilon \left(t\right).$$

We found the best kernel by building a design matrix *D* of lagged copies of *d*(*t*) and running the MATLAB instruction *k* = *D*\*G*. Kernels were fit based on 80% of the data, and the performance of each kernel was tested on the remaining 20% (cross-validation). For each experiment, we calculated the prediction quality *Q* of the linear model prediction as the cross-validated fraction of explained variance, averaged over the validation sets. To obtain an overall kernel across animals, we performed a weighted average across experiments, with weights given by the number of trials in each experiment.

## RESULTS

**Fig. 1.**
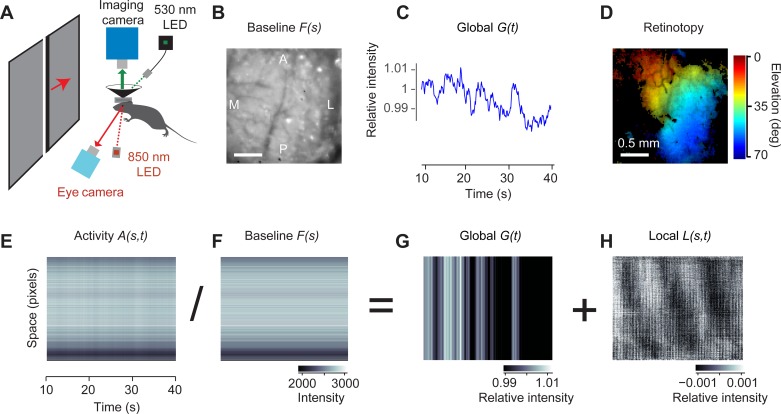
Separating hemodynamic activity in mouse visual cortex into local and global components. *A*: experimental setup showing the camera for intrinsic imaging and the green LED (530 nm), the camera for eye tracking and the infrared LED (850 nm), the monitor showing the drifting bars, and the head fixation system. *B*: the spatial component *F*(*s*) of the separable model (*Eq. 1*). A, anterior; P, posterior, L, lateral, M, medial. *C*: the temporal component *G*(*t*) of the separable model (global component). *D*: map of preferred elevation obtained from the data in *H*. *E*: the response to moving flickering bars, expressed as a function of space (concatenated into a single dimension) and time, 10–40 s after stimulus onset. *F*: the baseline image in *B* expressed as a 2-D matrix. *G*: the global component in *C* expressed as a 2-D matrix. *H*: the local component. Pixels in *E–H* were sorted by their phase at the frequency of the stimulus.

To measure hemodynamic activity, we performed intrinsic optical imaging in mouse posterior cortex in a region that included area V1 (Fig. 1). Mice were held head-fixed and were allowed to adjust their body position as desired. We illuminated the cortex with a green light at 530 nm (Fig. 1*A*), which reveals variations in blood volume, a measure closely related to neural activity (Sirotin et al. 2009). To elicit activity in visual cortex, we used a stimulus that is widely used to map retinotopy (Kalatsky and Stryker 2003): a flickering bar drifting slowly and periodically (0.10–0.12 Hz) across the contralateral visual field.

Meanwhile, we imaged one eye with a camera under infrared light, to reveal eye movements and pupil dilations. Consistent with previous measurements (Sakatani and Isa 2007), large eye movements (>1°) were rare, occurring about every 6 s (0.16 ± 0.09 times per second, mean ± SE, *n* = 26 experiments in 12 mice), and were mostly horizontal (5.3 ± 0.6° in azimuth vs. 2.4 ± 0.3° in elevation).

### 

#### Separating local and global components of hemodynamic activity.

To analyze the resulting hemodynamic activity, we expressed the 3-D data (rows × columns × time) as a 2-D matrix with coordinates space and time (Fig. 1*E*). This matrix was dominated by horizontal stripes, which arise because some pixels are darker than others. These were modulated by faint vertical stripes, which represent global activity that invests the whole image synchronously.

To separate these modulations, we fitted this matrix with a space-time separable model, the product of a map of space, *F*(*s*), to capture the horizontal stripes, and a global function of time, *G*(*t*), for the vertical stripes:

(1) A(s,t)=F(s)G(t)+R(s,t).

We obtained the optimal *F*(*s*) and *G*(*t*) values by minimizing the residual, *R*(*s*, *t*), i.e., the portion of the data that is not explained by the model. Because *F*(*s*) and *G*(*t*) multiply each other, their relative scaling is ambiguous. To resolve this ambiguity, we set *G*(1) = 1. The space-time separable model *F*(*s*)*G*(*t*) accounted, on average, for 94.6 ± 0.4% of the total variance in the data (*n* = 42 experiments in 13 mice), capturing variance both across space (Fig. 1*B*) and across time (Fig. 1*C*).

As expected, the function of space *F*(*s*) was an overall image of the baseline intensity of the cortex, with some pixels darker than others due to uneven illumination and the different reflectance of cortex and blood vessels (Fig. 1*B*). When replicated over time, this image recapitulated the horizontal stripes seen in the data *A*(*s*, *t*), i.e., those aspects of that activity that were constant across time (Fig. 1*F*).

We could thus use this baseline image *F*(*s*) to divide the imaged data and observe its fluctuations relative to baseline (Fig. 1, *E–H*). This procedure is similar to dividing each pixel by its value in the first frame or by its time average, which are standard practices in optical imaging (Bonhoeffer and Grinvald 1996). Performing this division essentially removes the horizontal stripes from both the separable model and the residual. The resulting relative response is therefore decomposed into two components, one global and one local:

(2) $$\frac{A\left(s,\;t\right)}{F\left(s\right)}=G\left(t\right)+L\left(s,\;t\right).$$

The global component, *G*(*t*), is simply the global function of time obtained in the previous equation. It reflects variations in hemodynamic activity that invest simultaneously the whole image, and it is an apparently erratic trace (Fig. 1, *C* and *G*). The local component, *L*(*s*, *t*), in turn, is a pixel-by-pixel rescaling of the residual in the previous equation, *R*(*s*, *t*)/*F*(*s*). It depends not only on time but also on space. By definition, it contains all activity that changes at different times in different places in cortex.

This local component was clearly periodic, reflecting the presumed neural response to the periodic stimulus (Fig. 1*H*). The stimulus causes neural activity to be organized in travelling waves, whose phase could be used (Kalatsky and Stryker 2003; Pisauro et al. 2013) to obtain characteristic maps of retinotopy (Fig. 1*D*). Indeed, we chose a moving visual stimulus precisely because, due to the retinotopic organization of visual cortex (Fig. 1*D*), it drives responses in different cortical locations at different times (Fig. 1*H*).

#### The global component is larger than the local component and is not stimulus driven.

For the purposes of measuring stimulus-driven or task-related activity, the global component is often considered as a form of noise (see discussion). By definition, indeed, global activity occurs simultaneously in different areas and is widely distributed across the cortex. The visual stimuli, by contrast, would drive only the visual cortex, and their movement would engage its different retinotopic regions at different times.

**Fig. 2.**
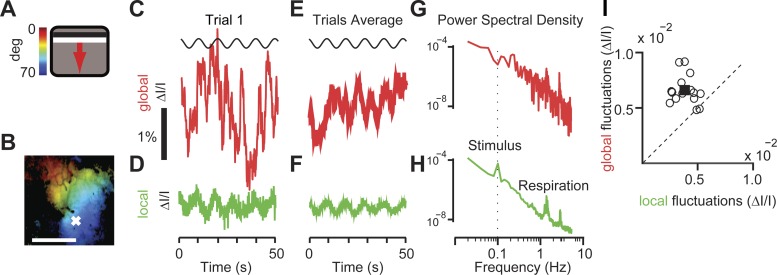
Local activity, unlike global activity, reflects sensory responses. *A*: moving bar stimulus (4° wide drifting at 0.12 Hz for 5 cycles) and color bar mapping degrees of visual field to the position of the bar on the screen. *B*: map of retinotopy obtained through frequency analysis of the local component. Colors indicate preferred position in visual field (color scale in *A*). Brightness indicates signal amplitude. Scale bar, 1 mm. *C*: time course of global activity (Δ*I*/*I*) for a single stimulus presentation. *D*: same as *C* for the local activity measured in the representative pixel marked with a white cross in *B*. *E* and *F*: same as *C* and *D* but averaged across repeats. *G* and *H*: power spectral density of the global component and local component. A peak at frequencies of ∼2–3 Hz reflected respiration rate. Dotted line indicates the frequency of the stimulus. A peak was only present in the local component. The power spectral density of the local component was averaged across all pixels. *I*: average amplitude of the fluctuations for 17 experiments for the global (*y*-axis) and local (*x*-axis) components. Each circle represents an individual experiment. The square indicates mean across the population.

Indeed, the responses to the visual stimuli were largely contained in the local component and essentially absent from the global component (Fig. 2). We considered responses to a bar drifting slowly across the contralateral visual field and cycling across the screen with a period of 8.3–10.0 s (Fig. 2*A*). We then selected a point in the retinotopic map (Fig. 2*B*) and compared its local activity with the global component. As expected, the local activity was periodic, oscillating with the same period as the stimulus (Fig. 2*D*) and becoming clearer after being averaged over five trials (Fig. 2*F*). Indeed, its power spectrum contained a clear peak at the stimulus frequency, 0.12 Hz (Fig. 2*H*). The global component, instead, showed erratic, large fluctuations in individual trials (Fig. 2*C*) that were greatly reduced when averaged across trials (Fig. 2*E*) and contained little power at the frequency of the stimulus (Fig. 2*G*).

The temporal fluctuations in the global activity, however, were considerably larger than those in the local, sensory-driven activity (Fig. 2*I*). To measure the local stimulus-driven fluctuations, we computed the standard deviation of the stimulus-driven local activity (obtained by averaging across trials, as in Fig. 2*F*), and we averaged the results across pixels and stimuli. To measure the global fluctuations, we computed the standard deviation of the global activity (e.g., Fig. 2*C*). The global fluctuations were almost twice as large as the local stimulus-driven fluctuations (Fig. 2*I*): the local stimulus-driven fluctuations averaged 4.0 ± 0.9 (Δ*I*/*I* × 10^−4^) and were only about 60% as large as global fluctuations, which averaged 6.7 ± 1.3 (Δ*I/I* × 10^−4^), a significant difference in amplitude (*P* < 0.001, *n* = 17 experiments in 10 mice).

#### The global component is tightly coupled to variations in alertness measured through pupil dilation.

The global fluctuations are large and are not driven by sensory input. Are they a form of noise, or do they reflect deterministic, nonsensory factors? Studies in primates, including humans, have revealed multiple nonsensory factors that can influence hemodynamic activity, including attention, response preparation, alertness, and task engagement (Cardoso et al. 2012; Fox et al. 2006; Jack et al. 2006; Ress et al. 2000; Serences et al. 2004; Sirotin and Das 2009). To determine if such factors might play a role in the global fluctuations, we measured pupil dilation. Pupil dilation is a reliable measure of alertness in multiple species, including mice (McGinley et al. 2015a, 2015b; Vinck et al. 2015), and has been related to hemodynamic activity in monkeys (Sirotin and Das 2009). We asked if there was a relationship between this measure and the global component of hemodynamic activity.

**Fig. 3.**
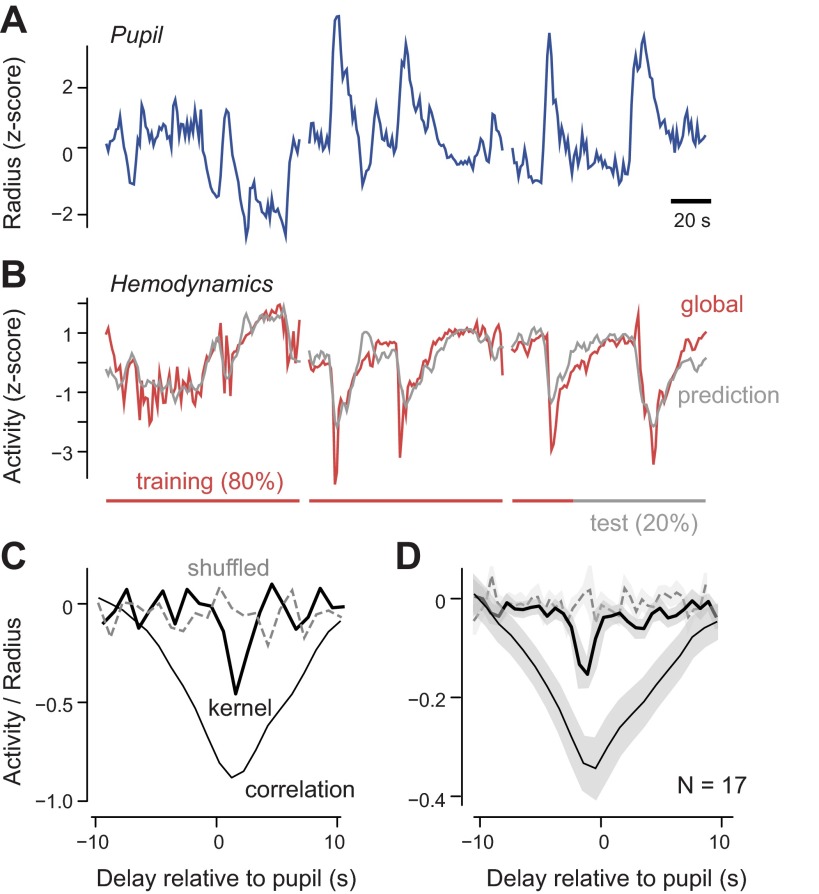
Relationship between pupil dilations and global hemodynamic activity. *A*: 3 consecutive recordings of pupil dilations. Traces are briefly interrupted between recordings. *B*: simultaneously measured global fluctuations of the hemodynamic activity (red). The prediction for the global hemodynamics (gray) is obtained by convolving the kernel (black trace in *C*) with the pupil radius trace. The kernel is computed over a training set of 80% of the data (red bar) but also predicts the test data (black bar). All traces in *A* and *B* are *z*-scored and therefore dimensionless. *C*: correlation between pupil radius and global hemodynamics (thin black trace) and kernel predicting global hemodynamic activity computed from the trials in *A* and *B* (thick black trace) or computed by shuffling the trial number in the eye tracking experiment (gray dashed trace). *D*: average correlation between pupil radius and global hemodynamics (thin black trace) and average kernel predicting global hemodynamic activity from pupil dilations (thick black trace), in 17 experiments (*n* = 10 mice). When we shuffled the trial numbers in each eye-tracking experiment, the peak in the kernel disappeared (dashed trace).

During most trials, mice spontaneously dilated their pupils, and these dilations tended to last several seconds (Fig. 3*A*). The pupil area varied by a factor of 2.8 ± 0.3 between minimum and maximum. These dilations were sporadic. For instance, dilations larger than 20% from the mean occurred 1.4 ± 1.4 times per minute, with an average duration of 1.8 ± 0.1 s. These dilations were observed in conditions of constant illumination, so they did not reflect mechanisms that control optic flux. Moreover, they did not show an obvious relationship to the visual stimuli. They are likely to reflect increases and decreases in the animal's alertness (McGinley et al. 2015a, 2015b; Vinck et al. 2015).

These spontaneous dilations and constrictions of the pupil correlated with the global component of the hemodynamic signals (Fig. 3, *B–D*). Pupil dilations were accompanied by darkening of the cortex (Fig. 3*B*), which indicates an increase in absorbance, most likely due to increases in blood volume. Pupil radius and the global hemodynamic activity were indeed anticorrelated, both in the example experiment, where this relationship is particularly evident (ρ = −0.86; Fig. 3*C*), and across experiments, where it is also highly significant (ρ = −0.36 ± 0.06; Fig. 3*D*). This anticorrelation did not depend on visual stimulation, because it disappeared when we shuffled the trial numbers while keeping the stimuli constant (Fig. 3, *C* and *D*).

The strong correlation between pupil dilation and global hemodynamic signal had a broad temporal waveform, mainly because the individual signals varied slowly. To assess the relationship between the two signals (Fig. 3, *C* and *D*) on a finer temporal scale, we used linear regression and obtained the best-fitting filter (or “kernel”) that best predicted the global hemodynamic activity, once convolved with the pupil radius. This linear regression model yielded good fits (e.g., Fig. 3*B*). It accounted on average for 14 ± 4% of the variance of the global fluctuations (*n* = 17 experiments in 5 mice). This is a considerable fraction of the variance, given that it is cross-validated. It confirms the relationship revealed by correlation (Fig. 3*D*).

The resulting kernels were tighter in time than the measures estimated from correlation but were inconclusive about the delays between pupil dilation and global hemodynamic activity (Fig. 3, *C* and *D*). This delay was ∼0.5 s for the example data set (Fig. 3*C*) but varied between −0.5 and 0.5 s across data sets (Fig. 3*D*). At first sight, such short or negative delays may suggest a noncausal relationship: if the global hemodynamic fluctuations were due to an increase in neural activity triggered by the pupil dilation, there would be a delay between the two of close to 1 s (Pisauro et al. 2013) with the hemodynamic activity following and not preceding the dilation. However, if the delay involved in pupil dilation is also in the range of 1 s, then pupil dilation and global hemodynamic activity may both stem from a common cause.

## DISCUSSION

We have shown that the hemodynamic activity measured in the cortex of awake mice during visual stimulation can be readily separated into two components, one local and one global. The local component was restricted to portions of visual cortex and was tightly driven by the visual stimulus. The global component impacted simultaneously larger regions of cortex, including visual cortex and beyond, and was considerably stronger. Rather than being driven by stimuli, it was tightly coupled to spontaneous variations in alertness measured through pupil dilation.

These results contradict the common assumption that global hemodynamic activity is a form of hemodynamic noise. In traditional methods for the analysis of hemodynamic activity, global activity is “often discarded as a meaningless baseline” (Yang et al. 2014). It is typically subtracted or regressed away and then discarded. This approach is common both in optical imaging (Cheng et al. 2001; Grinvald et al. 1986; Schuett et al. 2002; Shtoyerman et al. 2000) and in fMRI (Aguirre et al. 1998; Birn et al. 2006; Fox et al. 2009; Glover et al. 2000; Lund et al. 2006; Macey et al. 2004; Wise et al. 2004; Zarahn et al. 1997).

Our results, instead, indicate that global component carries signals related to brain state, as reflected, for instance, in pupil dilation. In humans, pupil dilations correlate with several cognitive factors, including memory load, cognitive difficulty, valence, arousal, and task engagement (Beatty 1982; Wang 2011). In mice, pupil dilation is a reliable measure of alertness and correlates strongly with other indicators such as locomotion (McGinley et al. 2015a, 2015b; Vinck et al. 2015).

To separate local and global hemodynamic activity, we used a method that operates on all the pixels at once. An alternative approach would be to fit a model on a pixel-by-pixel case. A possible model of this kind would include a linear sum of a sinusoidal component related to the stimulus and a scaled version of the pupil. In pilot measurements, we found that this model performed well, but there were strong differences in fitted weights between the blood vessels and the nearby pixels, which require further investigation. Models of this kind could also be used to investigate possible multiplicative interactions between pupil dilation and stimulus responses.

Overall, our results seem consistent with previous optical measurements in visual cortex of awake monkeys (Cardoso et al. 2012; Sirotin and Das 2009). These measurements yielded traces that could be separated into two additive components. The first component was coupled with stimulus-driven local neural activity and thus resembles our local component. The second component, like our global component, was independent of the stimulus. Rather, it correlated with the temporal structure of the trial (Cardoso et al. 2012), with performance in the task, and with pupil dilation (Sirotin and Das 2009).

Moreover, the properties we have seen in the global component are consistent with several fMRI studies reporting nonsensory influences on hemodynamic activity in humans. These influences include attention (Ress et al. 2000; Serences et al. 2004), task structure (Cardoso et al. 2012; Donner et al. 2008; Jack et al. 2006; Sirotin and Das 2009), and coherent spontaneous activity (Fox et al. 2006; Yellin et al. 2015). Consistent with the idea that these influences would be global, their effects seemed to be spatially nonspecific or shared by large cortical regions. Also consistent with our results, trial-to-trial fluctuations in fMRI measurements in human frontal cortex were found to correlate with pupil dilations (Siegle et al. 2003).

Whereas the local component of hemodynamic activity appears to be tightly linked to driven neural activity, it is less clear whether the global component is due uniquely to neural activity. Extracellular recordings revealed no tight relationship between nonsensory hemodynamic activity and neural activity (Sirotin and Das 2009). Perhaps the main neural correlates of nonsensory factors take place below threshold and thus require intracellular measurements (McGinley et al. 2015a; Reimer et al. 2014). Another contribution to global activity may come from the noradrenergic system, which is thought to determine overall vascular tone, to be central in the maintenance of arousal, and to elicit pupil dilations (Gilzenrat et al. 2010; Joshi et al. 2016; Samuels and Szabadi 2008).

There remain a number of open questions regarding the interpretation of the global patterns and their possible use as a diagnostic tool. Indeed, the global signal may turn out to be useful to diagnose and investigate brain diseases such as schizophrenia (Yang et al. 2014). Distinguishing local and global contributions to hemodynamic activity thus may help us understand these diseases. More generally, it may improve our understanding of neurovascular coupling and our ability to interpret and design optical and fMRI measurements of brain responses.

## GRANTS

This work was supported by the Wellcome Trust and the European Research Council. M. A. Pisauro was supported by a University College London/Fight for Sight studentship. M. Carandini holds the GlaxoSmithKline/Fight for Sight Chair in Visual Neuroscience.

## DISCLOSURES

No conflicts of interest, financial or otherwise, are declared by the authors.

## AUTHOR CONTRIBUTIONS

M.A.P., A.B., and M.C. conception and design of research; M.A.P. and A.B. performed experiments; M.A.P. analyzed data; M.A.P., A.B., and M.C. interpreted results of experiments; M.A.P. and M.C. prepared figures; M.A.P. drafted manuscript; M.A.P., A.B., and M.C. edited and revised manuscript; M.A.P., A.B., and M.C. approved final version of manuscript.
